# Effects of sertraline in the prevention of low blood pressure in patients undergoing hemodialysis

**DOI:** 10.1590/2175-8239-JBN-2018-0189

**Published:** 2019-08-01

**Authors:** Christine Zomer Zomer Dal Molin, Thiago Mamoru Sakae, Fabiana Schuelter-Trevisol, Daisson Jose Trevisol

**Affiliations:** 1 Universidade do Sul de Santa Catarina, Tubarão, SC, Brasil.

**Keywords:** Renal Dialysis, Hypotension, Sertraline, Renal Insufficiency, Chronic, Diálise Renal, Hipotensão, Sertralina, Insuficiência Renal Crônica

## Abstract

**Introduction:**

Intradialytic hypotension (IDH) is a major complication of hemodialysis, with a prevalence of about 25% during hemodialysis sessions, causing increased morbidity and mortality.

**Objective:**

To study the effects of sertraline to prevent IDH in hemodialysis patients.

**Methods:**

This was a double-blind, crossover clinical trial comparing the use of sertraline versus placebo to reduce intradialytic hypotension.

**Results:**

Sixteen patients completed the two phases of the study during a 12-week period. The IDH prevalence was 32%. A comparison between intradialytic interventions, intradialytic symptoms, and IDH episodes revealed no statistical difference in the reduction of IDH episodes (*p* = 0.207) between the two intervention groups. However, the risk of IDH interventions was 60% higher in the placebo group compared to the sertraline group, and the risk of IDH symptoms was 40% higher in the placebo group compared to the sertraline group. Survival analysis using Kaplan-Meier estimator supported the results of this study. Sertraline presented a number needed to treat (NNT) of 16.3 patients to prevent an episode from IDH intervention and 14.2 patients to prevent an episode from intradialytic symptoms.

**Conclusion:**

This study suggests that the use of sertraline may be beneficial to reduce the number of symptoms and ID interventions, although there was no statistically significant difference in the blood pressure levels.

## Resumo

**Introdução:**

A hipotensão intradialítica (HID) é uma das principais complicações da hemodiálise, com uma prevalência de cerca de 25% durante as sessões de hemodiálise, causando aumento da morbimortalidade.

**Objetivo:**

Estudar os efeitos da sertralina na prevenção da HID em pacientes em hemodiálise.

**Métodos:**

Este foi um ensaio clínico duplo-cego, cruzado, comparando o uso de sertralina versus placebo para reduzir a hipotensão intradialítica.

**Resultados:**

Dezesseis pacientes completaram as duas fases do estudo durante um período de 12 semanas. A prevalência de HID foi de 32%. Uma comparação entre intervenções intradialíticas, sintomas intradialíticos (ID) e episódios de HID não revelou diferença estatística na redução dos episódios de HID (*p* = 0,207) entre os dois grupos de intervenção. No entanto, o risco de intervenções para HID foi 60% maior no grupo placebo em comparação com o grupo Sertralina, e o risco de sintomas ID foi 40% maior no grupo placebo em comparação com o grupo Sertralina. A análise de sobrevida utilizando o estimador de Kaplan-Meier corroborou os resultados deste estudo. A sertralina apresentou um número necessário para tratar (NNT) de 16,3 pacientes para prevenir um episódio de intervenção de HID e 14,2 pacientes para prevenir um episódio de sintomas intradialíticos.

**Conclusão:**

Este estudo sugere que o uso de sertralina pode ser benéfico para reduzir o número de sintomas e intervenções de HID, embora não tenha havido diferença estatisticamente significante nos níveis pressóricos.

## Introduction

Chronic kidney disease is currently considered a public health problem because of its high prevalence^1^^-^ 4. The number of patients suffering from end-stage renal disease (ESRD) is expected to rise even more in developing countries, as there is an increase in the elderly population, a higher prevalence of cardiovascular diseases and improvement in their treatment, as well as persistent high rates of hypertensive and diabetic patients^1^^,^ 5 ^-^ 7.

Hemodialysis (HD) is a widely used alternative therapy to treat ESRD patients^6^^,^ 8. HD imposes a great psychosocial overload on patients and their families and can be aggravated by the existence of comorbidities, such as diabetes mellitus, cardiovascular disease, peripheric neuropathy^3^^,^ 9, and by the complications during hemodialysis, such as hypotension, fatigue, cramps, headache, and others^10^^,^ 11.

Intradialytic hypotension (IDH) is the most frequent complication among hemodialysis patients^10^^-^ 14. Estimates suggest that up to 75% of patients suffering from ESRD experience one or more episodes of hypotension during hemodialysis within 6 months from initiation, with a prevalence of about 25% during hemodialysis sessions, depending on the definition used to describe IDH^13^.

Pathophysiology of IDH is complex and multifactorial^15^, involving different systems and measures, such as osmolarity drop, dialysate temperature, membrane biocompatibility, the use of acetate as buffer, and endotoxin entry into the circulation^10^^,^ 16 ^,^ 17.

The main mechanisms involved in hypotension during a hemodialysis session include plasma volume, volume preservation during ultrafiltration (UF), and cardiovascular compensation^16^.

Usually when there is a low UF rate, blood pressure (BP) is maintained during HD through capillary refill, peripheral vascular resistance and cardiac output^15^^,^ 18 ^,^ 19 Other factors like plasma osmolarity, autonomic dysfunction, and increased vasodilator synthesis are also related to this hemodynamic instability.^(15 )^

These events may lead to reduction of intravascular volume, increased release of vasodilatory substances, and reduction in vasoconstrictors, as well as complement activation and cytokine release. In turn, these mechanisms lead to reduction of cardiac output and peripheral vascular resistance, with consequent reduction of arterial pressure^16^.

Factors such as body weight, degree of hydration of the interstitial space, osmolarity, and concentration of plasma proteins and variations in regional blood flow distribution during dialysis also contribute to the hypotensive episode^16^.

Non-modifiable patient-related demographic factors include advanced age (≥ 65 years), female gender, Hispanic origin, and long dialysis^18^.

Comorbidities associated with increased risk of IDH include diabetic nephropathy, cardiovascular disease (including left ventricular hypertrophy, diastolic dysfunction, and systolic dysfunction. Patients with congestive heart failure and/or previous myocardial infarction), high weight gain between dialysis sessions (>3% of body weight), aneurysm patients and patients with autonomic dysfunction are at higher risk of developing IDH^18^^,^ 20 ^,^ 21. Other factors that contribute to IDH are: low blood pressure (BP) before the HD session (SPB less than 100 mmHg), hyperphosphatemia, meals before dialysis, and others^20^^,^ 22.

Patients on regular hemodialysis who have moderate to severe IDH have a higher prevalence of cardiovascular events, such as myocardial ischemia and myocardial dysfunction^7^; in addition, there are other mechanisms involved, including neurological damage and intestinal bacterial translocation. Imaging studies, such as magnetic resonance imaging of the skull, evidence cerebral ischemia, including cerebral infarctions, atrophy, and altered white matter, which is a risk factor for dementia and stroke. Bacterial translocation occurs due to organ edema and hypoperfusion, contributing to pro-inflammatory stimuli and aggravating the malnutrition of these patients^18^.

The prompt recognition by the hemodialysis team, associated with modifications in the patients’ dialysis therapy, has been adopted for the management and prevention of hypotensive episodes. However, despite these measures, IDH prevalence remains high^13^^,^ 14 ^,^ 17 ^,^ 18.

Previous studies have indicated that selective serotonin reuptake inhibitors have improved symptoms in orthostatic hypotension and neurocardiogenic syncope, and have been used as an alternative treatment in patients who develop intradialytic hypotension^23^^-^ 29. Ultrafiltration performed during hemodialysis seems to be the main factor for the activation of the sympathetic reflex and vasoconstriction, which has the purpose of preserving BP at normal levels. However, paradoxical inhibition of this pathway occurs due to the sudden increase of serotonin in the central nervous system, which causes hypotension and vasodilatation^24^.

Sertraline has been studied with the aim of improving the response to the sudden increase of serotonin by sympathetic inhibition. Furthermore, it is widely used in the dialysis population^30^^,^ 31 at low cost and with high safety due to its pharmacokinetics and pharmacodynamics, efficacy, tolerability, and very few drug interactions^24^. The hypothesis of this study was that the use of sertraline prevents hypotensive episodes during hemodialysis, with positive impacts on the quality of life, decrease in the number of interventions and interruptions of dialysis treatment, as well as improves patient compliance and laboratory test results.

Lastly, considering that previous reports on the effect of serotonin reuptake inhibitors in the management of IDH episodes still need to be confirmed by further studies^7^^,^ 32, a double-blind, crossover trial was carried out to compare sertraline and placebo and assess the medication efficacy on the prevention of IDH episodes in hemodialysis patients.

## Methods

A double-blind, crossover clinical trial was conducted to compare placebo and sertraline in reducing hypotension in patients undergoing hemodialysis. ESRD patients undergoing hemodialysis for at least 3 months were selected for the trial from a Hemodialysis Clinic of Southern Santa Catarina, Brazil^16^^,^ 33, from January to March, 2017.

The Open Epi software was used to calculate the sample size. For the statistical calculation, we used a 50% prevalence of exposed and 5% of unexposed treated subjects, with an 80% study power, sample size ratio of 1:1, which resulted in a total of 30 subjects, based on the worldwide prevalence of nephropathy, as well as hemodialysis patients with IDH^2^^,^ 15 ^,^ 16 ^,^ 18 ^,^ 20 ^,^ 32 ^,^ 34.

All patients underwent hemodialysis three times a week, with blood capillaries of biocompatible materials (polysulfone or cellulose triacetate), in hemodialysis machines, using a dialysate bath containing 35 mEq/L bicarbonate, 138 mEq/L sodium, 3.5 mEq/L calcium, 2 mEq/L potassium, and 1 mEq/L magnesium. The dialysate temperature was maintained at 37 degrees Celsius, the dialysate flow set at 500 to 600 mL/min and blood flow between 250 and 350 mL/min. Ultrafiltration volumes were removed constantly throughout the hemodialysis session.

The inclusion criteria were patients on hemodialysis three times a week, for at least three hours per day, and at least three months, aged 18 years or more, male or female according to previous study^35^. Patients should have presented intradialytic hypotension, characterized according to previous studies^25^^-^ 27 ^,^ 29 by the presence of an SBP decrease of at least 30 mmHg or pre-hemodialysis SBP less than or equal to 100 mmHg with any of the associated symptoms: headache, weakness, cramps, dizziness, blurred vision, nausea or vomiting, malaise; any SBP lower than 90 mmHg and/or DBP less than 40 mmHg; or symptoms previously mentioned that required intervention by the nursing team^25^^,^ 35.

Patients eligible for the study had IDH defined by the above criteria in at least 50% of the hemodialysis sessions of the last three months prior to the start of sertraline use^26^.

Patients with known hypersensitivity to sertraline, with known hemodynamic instability, such as systemic infection, or unfavorable clinical conditions, such as advanced liver cirrhosis and decompensated heart failure, were excluded.

Patients with acute renal failure or those who previously took antidepressants or serotonin reuptake blockers were excluded^25^^,^ 36.

All patients were also carefully evaluated for dry weight, clinical history, comorbidities, and if necessary, physical examination and chest X-ray to complement the common clinical examination. They were instructed not to take their antihypertensives before the sessions, to maintain their interdialytic weight gain (IDWG) between sessions, emphasizing salt and fluid restriction, according to the routine adopted among all patients in the Hemodialysis Clinic.

Previous studies have reported trials with randomization in two groups (placebo versus medication)^24^^,^ 28. In this study, all participants received only a placebo at the beginning of the study for a 6-week period. After that, they received sertraline for another 6 weeks, which meant that the patients were their own controls (crossover trial), with a week washout period. Patients and their families, as well as physicians (not involved in the research), nursing staff, and other key personnel directly involved in the patient’s care were blinded to the use of sertraline or placebo. Only study investigators were not blinded to placebo or sertraline use.

The sertraline capsules were obtained from a specialized laboratory after quality assurance through a certificate of analysis. The placebo capsules were manufactured and packaged in a similar way to the sertraline capsules by the same laboratory.

Pre- and post-dialytic dry weight, and need, number, and type of interventions performed during IDH episodes were evaluated. The laboratory tests were evaluated according to the Hemodialysis Clinic routine. During the hemodialysis sessions, blood pressure measurements were taken on at least three occasions to calculate mean arterial pressure^29^.

## Results

We selected 18 of 55 patients treated at the Hemodialysis Clinic of Araranguá, state of Santa Catarina, Brazil, at the time of the study, representing an IDH prevalence of 32%. Two of the 18 patients were excluded from the study. One requested his withdrawal because of his assistant cardiologist advice, and the other patient died of unrelated causes before starting the use of placebo.

The remaining 16 patients participated in both phases of the study (placebo and sertraline), completing 12 weeks of treatment. Regarding the demographic data, the sample was composed of 8 women and 8 men; 10 patients were white and 6 were non-whites. The mean age of study participants was 61 years (SD=15.73). 9 patients had diabetes mellitus and 7 suffered from hypertension.

With regard to vascular access, 15 patients underwent hemodialysis through an arteriovenous fistula and one underwent hemodialysis through a double lumen catheter.

As the present study used a crossover design, in which the cases were their own controls, the sample size was 32.

The results of the laboratory tests for the 16 patients are displayed in Table 1. Data represent the average of the three measurements of the exams collected during the study period.

**Table 1:** Lab test results of the study participants

	Minimum score	Maximum score	Mean	Standard deviation
Kt/V	1.4	1.6	1.54	0.058
URR	76	84	80.58	2.18
Ur pre	86	216	131.57	29.36
Ur pos	12	48	24.83	8.38
Ca	7.20	10.30	8.76	0.63
K	3.70	5.60	4.92	0.45
P	2.40	8.90	5.54	1.91
Ht	21.10	39.10	31.72	4.37
Hb	7.10	12.80	10.45	1.41
Vol UF	0.00	6.40	2.12	1.29
UF rate	0.00	1633.33	596.42	334.28
IDWG	0.00	7.90	2.14	1.42
Weight pre	48.85	112.60	76.12	15.32
Weight post	48.00	111.70	74.01	15.11

Kt/V

URR - Urea reduction ratio (%)

Ur pre - Urea pre-dialysis (mg/dL)

Ur pos - Urea pos-dialysis (mg/dL)

Ca - calcium (mg/dL)

K - potassium (mEq/L)

P - phosphorus (mg/dL)

Ht - hematocrit (%)

Hb - hemoglobin (g/dL)

Vol UF - ultrafiltrate volume (L)

UF rate - ultrafiltration rate (mL/H)

IDWG - interdialytic weight gain (kg)

Weight pre - pre dialysis session weight (Kg)

Weight post - post dialysis session weight (Kg)

The majority of patients had adequate examinations, and the laboratory abnormalities were those expected for nephropathic patients on hemodialysis. There was no statistically significant difference between the results of the tests during the use of placebo or sertraline. Thus, we present the data of the two groups together in Table 1.

A comparison between the placebo group and the sertraline group revealed a statistically significant difference (*p* < 0.05) between pre- (higher) and post-dialysis weight (lower). A significant difference (*p* < 0.05) was found in BP with a drop between pre- and post-hemodialysis in all SBP measurements (Table 2). A significant difference was found (*p* < 0.05) between the first and second DBP measurement, but not between the second and third (*p* = 0.75), as shown in Table 2.

**Table 2:** Description of interdialytic blood pressure measurements

Intradialytic blood pressure measurements	Mean (SD)	Confidence interval	*p*-value
SBP 1 X SBP 2	9.58 (23.72)	7.64 a 11.56	< 0.05
SBP 1 X SBP 3	7.74 (25.21)	5.65 a 9.84	< 0.05
SBP 2 X DBP 2	-1.82 (20.04)	-3.46 a -1.57	< 0.05
DBP 1 X DBP 2	4.08 (14.01)	2.91 a 5.24	< 0.05
DBP 1 X DBP 3	4.24 (14.05)	3.07 a 5.41	< 0.05
DBP 2 X DBP 3	0.14 (12.41)	-0.85 a 1.18	0.77

SBP - Systolic Blood Pressure

BDP - Diastolic Blood Pressure

Comparisons between intradialytic interventions, intradialytic symptoms, and IDH episodes revealed no statistical difference in the reduction of IDH episodes (*p* = 0.207) between the two intervention groups. However, the risk of IDH interventions was 60% higher in the placebo group compared to the sertraline group (RR = 1.59; 95% CI 1.03 to 2.48, *p* = 0.034). Likewise, the risk of ID symptoms was 40% higher in the placebo group compared to the sertraline group (RR = 1.42; 95% CI: 1.02 to 2.02, *p* = 0.038).

Sertraline presented a NNT of 16.3 patients to prevent an episode of IDH interventions and 14.2 patients to prevent an episode of ID symptoms.

**Graph 1:**
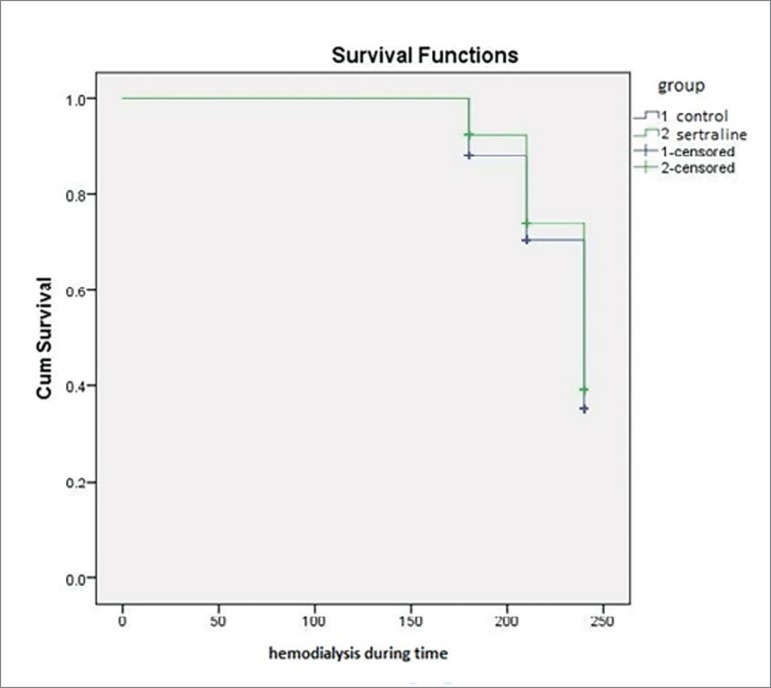
Survival analysis of the incidence of interventions and intradialytic hypotension episodes.

**Graph 2:**
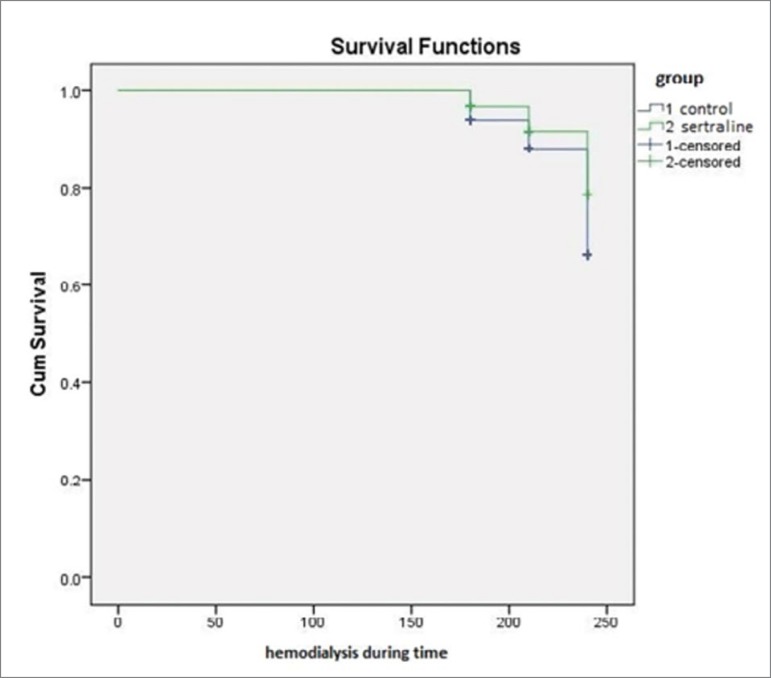
Survival analysis of the incidence of interventions and symptoms during hemodialysis.

Graphs 1 and 2 present a survival analysis by comparing the study groups with the incidence of IDH interventions (Graph 1) and ID symptoms (Graph 2). Kaplan-Meier survival analysis (time on hemodialysis was the time variable) presented in graphs 1 and 2 corroborated the results of the bivariate analysis, showing differences in the incidence of IDH interventions (log rank 4.38; *p* = 0.037) and ID symptoms (log rank 4.17; *p* = 0.041).

## Discussion

In the present study, there was a relatively high prevalence of patients with IDH (32%) compared to the findings of several studies, in which the prevalence was approximately 20%^2^^,^ 10 ^,^ 13 ^,^ 15 ^,^ 16 ^,^ 18 ^,^ 20 ^,^ 32. The high prevalence of IDH may be due to their selection during the summer in the southern hemisphere (January through March 2017), a season in which the patients ingest larger amounts of liquids, leading to an increase of the dry weight and IDWG, thus contributing to an eventual higher need for UF during the HD process. It is known that an UF of 10-13 mL/kg/h is not associated with increased mortality for most patients, except for those with heart failure^37^. Therefore, elevated IDWG generates higher UF rates, with a higher probability of IDH^14^^,^ 15. Furthermore, the mean age of the patients was 61 years and studies have shown a greater probability of IDH (up to 50%) among the elderly population^10^^,^ 32.

The majority of the patients in this study presented diabetes mellitus as a baseline disease, which was similar to the findings by Razeghi^24^, who found that 5 out of 12 patients had diabetes. This comorbidity is related to a greater number of cardiovascular complications^3^^,^ 9, and most of the studies that performed interventions excluded diabetic patients, as it was reported in the study by Yalcin and colleagues^27^ in which the incidence was 11% (12 patients out of 108 chronic kidney disease subjects had IDH), but patients with diabetes mellitus or autonomic neuropathy were excluded from the sample. Brewster^26^ has found an incidence of 26% of IDH using similar diagnostic criteria to our study. In a study by Tislér and colleagues^38^, patients who improved SBP in Trendelemburg position were not included as a frequent IDH subject, thus having a reduced incidence of about 10%.

In the present study, a larger number of white patients presented IDH compared to non-whites, which corroborated most studies that found higher hypertension rates in non-white patients^39^^-^ 41.

One patient had double lumen catheter access and was not excluded from the present study, since there was no evidence of catheter infection or other alterations that could contribute as bias. In addition, the patient had vascular access failure, a case in which hemodialysis by AVF is difficult.

No significant difference was found among laboratory tests taken in three occasions, during the study 12-week period. However, when the laboratory tests were evaluated individually, an improvement was found in the parameters, especially in the anemia control tests, reported as hemoglobin/hematocrit ratio (Hb/Ht). The improvement in these parameters may be attributed to the dialysis adequacy associated with medication dose adjustments, such as erythropoietin and intravenous iron.

In the present study, there was a statistical difference (*p* < 0.05) between the pre- and post-dialysis weight correlation, probably due to the fact that patients presented adequate UF during the hemodialysis session. SBP declined from the first to the second and from the second to the third measurement (Table 2). This finding corroborates the IDH presence among the study participants, which is in agreement with the findings of several studies that use SBP to assess IDH^15^^,^ 18 ^,^ 24 ^-^ 27 ^,^ 29.

Nonetheless, the comparison between the placebo and sertraline groups regarding the DBP levels, revealed a significant difference (*p* < 0.05) from the first to the second DBP measurement, and from the first to the third DBP measurement, whereas no statistically significant difference (*p* = 0.75) was found from the second to the third DBP measurement. This indicated that the diagnosis of IDH should be made through SBP level and drop, as well as by analyzing the mean arterial pressure decline using DBP as a factor^14^^,^ 37.

A statistically significant difference was found between heart rate and respiratory rate measurements in the pre- and post-hemodialysis period. No reported findings indicate a correlation between vital signs and IDH, but we observed a significant difference that could be explained by the hemodynamic response to the BP drop using the formula BP = CD x PVR, in which CD is the cardiac output and PVR the peripheral vascular resistance^42^. However, the respiratory rate (RR) and heart rate (HR) were not evaluated during the hypotensive episode itself, but in the pre- and post-hemodialysis periods. Consequently, the presence of a compensatory hemodynamic response to the BP drop or the absence of bradycardia could not be verified in those patients with paradoxical sympathetic reflex inhibition^10^^,^ 14 ^,^ 15 ^,^ 43.

Sertraline presented an NNT of 16.3 patients to prevent an episode of IDH interventions, and 14.2 patients to prevent an episode of ID symptoms. According to a meta-analysis by Cipriani and colleagues^44^ in which a comparison between SSRIs (sertraline vs. fluoxetine) was made for the treatment of major depressive disorder, sertraline was superior to fluoxetine, with an NNT of 12. This result can be attributed to the antidepressant effect of the medication, as reported in the study by Dheenan^29^. Based on that study and the NNT of this study, the clinical use of sertraline is supported.

Yalcin^25^^,^ 27, Dheenan^29^ and Razeghi^24^, who have described a significant effect in the control of IDH, support the use of sertraline as an alternative to other medications or techniques for the treatment of ESRD patients mainly because of its low toxicity, although the use of sertraline is not yet established as a therapeutic alternative to minimize the IDH disorder.

The present study showed that the blockade of serotonin reuptake is not efficient to minimize hypotension episodes during hemodialysis; however, it decreased the incidence of IDH interventions and ID symptoms, corroborating other studies, such as the one by Razeghi^24^, in which the number of interventions was reduced.

The use of higher doses of sertraline, as shown in a study by Yalcin^25^ in which the daily dose was 100 mg, revealed different findings than those of our study, which can be explained by the following sampling differences: patients selected for psychiatric illnesses and using antidepressants^38^ and exclusion of patients suffering from autonomic neuropathy or diabetes mellitus^25^^,^ 27 ^,^ 29.

Dheenan and colleagues^29^ have reported that the antidepressant effect could cause a decrease in complaints and demand for nursing interventions. Perhaps the antidepressant effect of sertraline improves the patients’ quality of life, well-being, symptomatology and tolerance and, consequently, reduces complaints in the intradialytic period. Some surveyed patients reported at-home improvements in the post-dialysis period, despite the unchanged blood pressure levels.

Data from the literature have indicated that sertraline takes four to six weeks to reach the maximum effect^24^^,^ 45. Most of the studies have analyzed a 4-week period of sertraline use^24^^-^ 27, and made suggestions to use it for a longer period to obtain better results and improve blood pressure levels. Dheenan^29^ has used sertraline during 6 to 12 weeks, with a 6-week monitoring period, so, in our study, we opted to use each drug for a 6-week period. Furthermore, there were reports of studies being affected by the washout period, which was avoided in our study, since we administered placebo to all patients as the first drug. However, despite the precautions taken from other studies, there was no improvement in BP levels during the 6-week period of sertraline use compared to placebo.

The main limitation of the present study (and other previous studies) is the small number of patients in each group. Nonetheless, the sample size was adequate, based on previous studies, for the validity of the results.

We used a crossover study design because of the advantage of allowing the use of smaller samples, since the cases are their own controls.

## Conclusion

The present study showed that the number of IDH episodes per session was similar for patients using either sertraline or placebo. It also showed that demographic data, laboratory tests, IDWG, and pre- and post-dialysis weight did not present a significant difference between the placebo and sertraline groups, with only a slight improvement in anemia control in the sertraline group.

Although no difference was found between the placebo and sertraline groups in blood pressure levels, there was an improvement in patients’ symptoms (even in those with IDH) as well as in the number of medical and nursing interventions during the intradialytic period, with a risk of IDH interventions 60% higher in the placebo group. The risk of ID symptoms, which was 40% higher in the placebo group compared to the sertraline group, was also significantly different. It would be necessary 16.3 patients using sertraline to prevent an episode of IDH intervention and 14.2 patients to prevent an episode of ID symptoms. This finding indicated that the sample size was adequate to test the efficacy of sertraline used in clinical practice.

In conclusion, sertraline may be beneficial in decreasing the number of ID symptoms and interventions. Further studies should be conducted to examine its antidepressant effect and efficacy for IDH treatment. Future studies should consider a longer period of patient monitoring after starting the use of sertraline, e.g., twelve weeks instead of six, and a larger number of participants for a better analysis of the hypotensive episodes.

**List of abbreviations**
IDH: Intradialytic hypotension
SSRIs: selective serotonin reuptake inhibitors
BP: blood pressure
SBP: systolic blood pressure
DBP: diastolic blood pressure
MAP: mean arterial pressure
ID: intradialytic
NNT: number needed to treat
ESRD: chronic end-stage renal disease
HD: hemodialysis
UF: ultrafiltration
IDWG: Interdialytic weight gain
SD: standard deviation
DM: diabetes mellitus
AVF: arteriovenous fistula
Hb: hemoglobin
Ht: hematocrit
CD: cardiac output
PVR: peripheral vascular resistance
RR: respiratory rate
HR: heart rate
